# Best practice in nurse-led chemotherapy review: a position statement from the United Kingdom Oncology Nursing Society

**DOI:** 10.3332/ecancer.2012.263

**Published:** 2012-07-31

**Authors:** E Lennan, C Vidall, H Roe, P Jones, J Smith, C Farrell

**Affiliations:** 1 University Hospital Southampton NHS Foundation Trust, Tremona Road, Southampton, SO16 6YD, UK; 2 Clinical Risk and Practice Development, Healthcare at Home, Bristol, UK; 3 North Cumbria University Hospitals NHS Trust (Carlisle & Whitehaven), Cumbria, UK; 4 Greater Midlands Cancer Network, Wolverhampton, UK; 5 Borders General Hospital, Melrose, UK; 6 The Christie NHS Foundation Trust, Manchester, UK

## Abstract

This position statement has been formulated by nurses from the United Kingdom Oncology Nursing Society (UKONS) to provide guidance on the provision of nurse-led chemotherapy review clinics for adult patients. For the purposes of this statement, a nurse-led chemotherapy clinic is defined as one that conducts formal review (in a consultation room) before the decision to proceed and prescribe the next cycle of chemotherapy. This statement does not address the toxicity checks that take place immediately prior to the administration of chemotherapy, although many of the same principles will apply to both settings.

## Background

It is over a decade since *The NHS Cancer Plan* sets out the requirement for ‘new ways of working to streamline cancer services around the needs of the patient’ [1]. The plan identified extended roles for nurses as one of the means for achieving reforms to cancer care and noted the increasing specialisation of the cancer nurse workforce. The commitment to the new NHS roles and new ways of providing cancer care was reiterated in 2007 in the *Cancer Reform Strategy *[2]. Also, *Cancer in Scotland: Action for Change* called for improved focusing of nursing expertise to ‘maximise opportunities for development of services’ [3].

The focus on extended roles for nurses is not unique to cancer, and there is a strong history of nurse-led care in fields such as respiratory health, diabetes, and heart disease [4]. In 2010, the Prime Minister’s independent commission on nursing and midwifery highlighted the role of nurses as ‘clinicians, managers, leaders, teachers, researchers, scholars, and policy-makers’ [4]. The commission also stated: ‘More direct access to nurse-led services would improve cost effectiveness and health outcomes, and remove system blockages that delay appropriate care [4]’. Similarly, a systematic review has concluded that interventions provided or led by nurses have a beneficial impact on outcomes in the management of long-term conditions [5]. Looking specifically at the management of patients with cancer, the systematic review states that nurse-led care can be more beneficial than doctor-led care in terms of physical outcomes, patient satisfaction, and care organisation, and that specialised cancer nursing improves patients’ ability to cope with their cancer [5]. Crucially, the report notes that long-term survival is not impaired by nurse-led care, compared with the traditional medical model [5].

The areas in which cancer nurses have extended their roles include nurse-led chemotherapy review. In these clinics, appropriately skilled nurses see patients scheduled for chemotherapy who have already been assessed by a doctor and who meet the local criteria for nurse-led review. The growing move to nurse-led (and pharmacist-led) chemotherapy review received a resounding endorsement in 2010 from the NHS National Cancer Action Team (NCAT) for England, which reported benefits such as increased capacity, reduced waiting times, and the opportunity to deliver care close to patients’ homes [6]. NCAT also made the following unequivocal recommendation:

All cancer networks and the providers of chemotherapy services should urgently assess the potential for nurse-led or pharmacist-led chemotherapy and agree appropriate working protocols.

The establishment of nurse-led chemotherapy review was somewhat *ad hoc*, involving a steep learning curve and several hurdles—not least the difficulties faced by many nurses seeking administrative support for their clinics. On the positive side, because nurse-led chemotherapy review had already been shown to be as effective as doctor-led chemotherapy review in various therapeutic areas [7–10], chemotherapy nurses were able to learn from their colleagues’ previous experiences.

Current nurse-led chemotherapy review follows a variety of different models (see ‘Clinic examples’ section). For example, some cover a wide range of malignancies and chemotherapy agents, while some are dedicated to a single treatment for one specified cancer. Some take place in parallel with (and geographically adjacent to) a medical consultant clinic, while some may run without an oncologist on site, although consultant support must always be readily accessible. Access to non-medical prescribing also varies from clinic to clinic. Depending on local arrangements, nurses may be able to: modify or delay a chemotherapy cycle within guidelines, manage supportive treatments such as antiemetics, interpret test results, and refer patients directly to other services such as imaging, palliative care, or mental healthcare.

Clearly, there is no one-size-fits-all nurse-led chemotherapy review model appropriate for adoption nationwide. However, certain facets will be universal. Most notably, nurses should not seek to duplicate the medical model or provide a stand-alone service. Instead, nurse-led chemotherapy review provides an opportunity to add nursing values to the wider chemotherapy service, in the form of, for example, holistic care and family support, complemented by the aspects of advanced nursing practice, such as patient examination, toxicity assessment, and, where possible, access to non-medical prescribing within the clinic team. Supervision by and communication with consultant colleagues are essential.

In addition, the lead chemotherapy nurse should be responsible for leading (and seeking out the necessary expertise for) the preparation and regular updating of a business plan, while all nurses who are (or will be) involved in the service will need awareness and understanding of the business plan and an opportunity to contribute ideas in the interests of ensuring sustainability.

This UKONS position statement sets out the core issues for nurse-led chemotherapy review for adult patients.

## Nurse-led chemotherapy review: responsibilities

- Ensuring safe, effective delivery of chemotherapy and co-medications to patients, and appropriate follow up/monitoring, according to the locally agreed protocols and pathways, in a manner that ensures equity of access and care.
- Provision of education, information, and advice for:
  - Patients and carers regarding cancer and its treatment, including clear guidance on how to report problems and/or seek advice outside of clinic hours.
  - Non-oncology-specialist healthcare professionals regarding cancer management, care pathways, and patient support.

- Provision of support for the patients’ families.
- Acting as the patients’ advocate in local policy/decision-making.
- Provision of a safe working environment for clinic staff.
- Ongoing assessment of the service provided, through audit, monitoring, and review:
  - Patient-care experience,
  - Cost-effectiveness and financial considerations [11, 12],
  - In addition, the lead chemotherapy nurse will be responsible for the ongoing safety and sustainability of the service through mentoring and development of staff, and active involvement in the business planning process.

## Skills required

Currently, there is lack of clarity regarding the competency requirements for nurses with extended roles [13], and the Nursing and Midwifery Council proposes a registerable advanced nurse practitioner role with established standards of knowledge and expertise. In the mean time, lead chemotherapy nurses (those heading nurse-led chemotherapy review services) are likely to offer a varied array of advanced skills and competencies when they embark on the role and develop along a continuum of expertise, with a view towards a future gold standard for educational attainment as the role becomes increasingly established. In a recent overview of nurse-led cancer clinics, the authors specifically recommend developing and supporting training initiatives in advanced practice [14].

Key skills and knowledge areas for nurse-led chemotherapy review are listed below. In addition, certain essential and recommended educational attainments are set out in Table 1:

- Knowledge (regularly updated as part of continued professional development) of the relevant disease areas and the anticancer medications used in the clinic, to include:
  - Clinical indications of the agent/agents used, singly and (where applicable) in combination,
  - Storage and handling requirements of individual agents,
  - Treatment administration, including any special precautions and/or co-medications required,
  - Associated toxicities.

- Awareness and application of appropriate local and national guidelines,
- Holistic patient assessment:
  - General health,
  - Activities of daily living, using established tools, e.g. the Instrumental Activities of Daily Living Scale [15],
  - Suitability for treatment, using established tools, e.g. World Health Organization (WHO) or Eastern Cooperative Oncology Group (ECOG) performance status scales [16],
  - Requirements for supportive or palliative care [17],
  - Signs/symptoms related to treatment administration, e.g. hypersensitivity reactions,
  - Signs/symptoms related to treatment toxicities (short and long term),
  - Psychological and social issues relating to cancer and its treatment.

- Holistic patient management:
  - Taking patients’ written consent for treatment according to the clinical guidelines [18] and local trust policy,
  - Delivery of chemotherapy, co-medications and supportive treatments according to local protocols,
  - Review and management of adverse events, including recognition and appropriate management of toxicities requiring urgent intervention,
  - Modification of chemotherapy, where indicated, and use of supportive medications,
  - Ongoing provision of information and education for patients and carers on the disease, its treatment and the likely adverse events, enabling (i) self-care where safe and appropriate; (ii) reporting of toxicities to the appropriate healthcare professional with due urgency,
  - Provision of appropriate advice and support for patients self-administering oral chemotherapy in their own homes, (see UKONS position statement on oral chemotherapy [19]),
  - Non-medical prescribing—preferred but not essential (see Prescribing issues).

- Effective communication with:
  - Patients, families, and carers (also when chemotherapy is complete, to ensure the patient does not feel ‘abandoned’),
  - Healthcare professionals, e.g. the multidisciplinary team, the wider chemotherapy service, the wider nursing team, consultant colleagues, and, in particular, oncology pharmacists,
  - Healthcare providers, e.g. the cancer network, the acute trust, the local acute oncology service, primary care, and the private sector,
  - Non-healthcare agencies, e.g. social services.

- Audit skills, to include clinical data collection, financial assessment, and use of nurse-sensitive outcome indicators [20],
- Critical reading skills.

In addition to these core skills, the nurse will lead the business case for the clinic and will seek the support and expertise of managers in the creation and delivery of a clear business plan:

- Before initiation of the service,
- If any major changes are proposed.

Nurses who are (or are likely to be) part of the proposed service or affected by any of the major changes need to be able understand and contribute to the business plan and identify potential problems and pitfalls, particularly from the viewpoints of service provision and patient care.

For specific national recommendations on chemotherapy competency issues in the different countries of the UK, please refer to the appropriate websites [21–24].

## Prescribing issues

### Non-medical prescribing

The advent and development of non-medical prescribing by nurses have helped to promote the development of nurse-led care [25]. Although the presence of a nurse prescriber within the clinic team is not a prerequisite for nurse-led chemotherapy review, it can add to the value of the nurse-led review through:

- Avoiding the delays inherent in a model whereby a doctor’s signature must be sought, e.g. for dose modifications (particularly where an appropriate doctor is not always present, e.g. in outreach services),
- Enhancing nurse involvement in decisions around the introduction of innovative treatments,
- Involving the nurse in the decision-making and application processes for access to drugs via the Cancer Drugs Fund (in England),
- Transparency of the professional development of nurses and their contribution.

Looking to the future, UKONS regards the availability of non-medical prescribing within the clinic as a gold standard for nurse-led chemotherapy review. Where non-medical prescribing is not already in place in nurse-led clinics, trusts should plan for a move towards its implementation when considering service development.

Clearly, a nurse who is a prescriber must be accountable for all related decision making and for maintaining competence [26]. Details of the legislative and professional issues surrounding non-medical prescribing by nurses are covered in detail elsewhere [27–29]. The authors note that, for safety reasons, a nurse who prescribes chemotherapy for a particular patient must not also administer the treatment.

Note that patient group directions (PGDs) are not permitted for chemotherapy agents but may be appropriate in the NHS for supportive medications. In addition, PGDs cannot be used in the private sector unless care is being provided for NHS patients from an NHS trust where the PGDs already exist. Where PGDs are used, the nurse must be fully aware of the requirement to complete the competency framework from the National Prescribing Centre [30].

In future, nurse prescribing is likely to be a key feature of nurse-led chemotherapy services, alongside other aspects of advanced practice, and UKONS actively encourages non-prescribers to consider completing an appropriate prescribing course.

### Electronic prescribing

Use of electronic (i.e. not paper based) prescribing of chemotherapy is specified by NCAT [6] and represents a future minimum standard for chemotherapy prescribing in all settings, including nurse-led clinics. Where trusts have not yet established electronic prescribing, computer-generated prescriptions will be essential. UKONS endorses the recommendation from the National Confidential Enquiry into Patient Outcome and Death [31] and the National Chemotherapy Advisory Group [32] that the patient record for prescribing should include a performance status record and a full toxicity record with agreed parameters (e.g. to ensure that chemotherapy is not delivered without appropriate assessment and intervention).

## Administrative and other support

In the experience of UKONS members, one of the hurdles faced by nurses embarking on leadership of clinical services is the presumption that they do not need administrative and in-clinic support. However, such support is essential since the nurses will have other pressures on their time. Therefore, if looking to establish nurse-led chemotherapy review, we recommend careful consideration of the staff required to fulfil the following tasks:

- Booking and organisation of clinic rooms,
- Appointment booking,
- Obtaining patients’ notes,
- Letter writing and routine telephone calls,
- Organisation of transport, where required,
- Calling patients in from the waiting area,
- Preparing the consulting room between patients, e.g. the examination couch etc.,
- Undertaking procedures such as venepuncture,
- Coding of activity (for the tariff).

## Audit and monitoring

It is essential to monitor the efficacy of nurse-led chemotherapy review through peer review, audit of outcomes (validation), and by conducting surveys of both patient and staff satisfaction with the service provided. Careful attention must be paid to the findings of such exercises, which should underpin ongoing service development. Repeat audits, surveys, etc. are an essential component of service review.

It is important to monitor and evaluate the whole chemotherapy service, including medical consultant-led clinics—i.e. not only the nurse-led clinic—to ensure continued efficacy, patient satisfaction, and a high standard of joint working across the service.

UKONS recommends nurses to conduct and assume accountability for their own service audits.

In the national arena, NCAT is currently developing a survey of patients’ experiences of chemotherapy, with the aim of obtaining information and data that will inform and support the shaping of future services.

## Financial considerations

Before endorsing the establishment of nurse-led chemotherapy review, the trust will need to see a business plan, setting out the rationale for the clinic, including:

- Details of current pressures such as availability of specialist nursing staff, shortage of appropriate medical staff, and lengthening patient waiting times,
- Details of forthcoming pressures, such as availability of newly approved treatments and increased use of non-traditional treatment delivery methods, e.g. oral chemotherapy,
- Contingency planning for annual leave, staff sickness, and other issues affecting staff availability.

**Figure 1:**
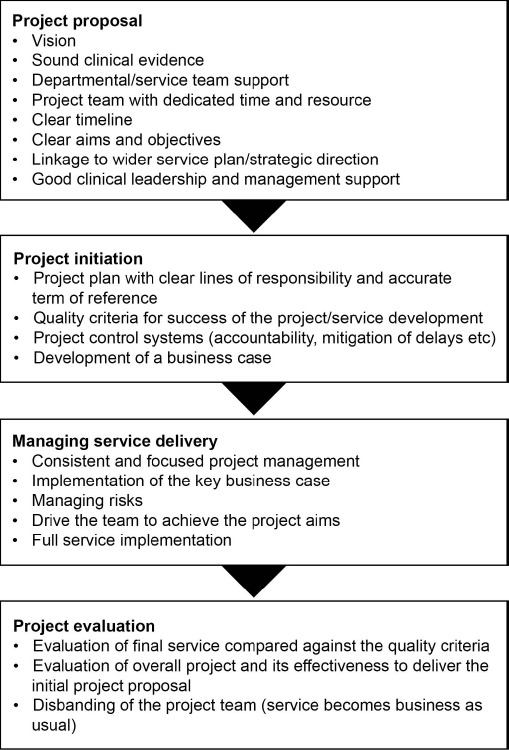
Key steps in the business for a new service (adapted from Orchestrate-online) [33].

An outline of the key issues to be addressed when presenting the plan for a new service, such as a nurse-led clinic, is shown in Figure 1 [33].

Once nurse-led chemotherapy review is established and running, all clinical activity will need to be logged and coded, to demonstrate the ongoing value of the nurse-led service and to ensure payment of any revenue earned by the service. The lead chemotherapy nurse will be responsible for making sure such data are collected and used appropriately.

## Information sharing

When leading chemotherapy review, the nurse has a duty to ensure collection and appropriate sharing of information, for example:

- Data required by cancer registries:
  - Ensure clarity on the datasets required.

- Data and other information for clinical trials (for participating clinics):
  - Ensure compliance with all requirements of clinical trials, e.g. compilation of log sheets and sign off by the principal investigator.

- Sharing of best practice locally and further afield through formal and informal educational events and publications.

## Clinical governance

Nurse-led chemotherapy review must have systems in place to ensure that nationally recommended protocols and polices for care quality and safety are developed, implemented, and audited at regular intervals [6, 31]. In addition, the overarching audit of the whole chemotherapy service should seek to ensure that nurse-led chemotherapy review does not in any way impair the quality of the service received by other patients.

## Other key issues

- Provision of nurse-led chemotherapy review is not precluded by the treatment setting, e.g. cancer centre/unit, outreach clinic, or the patient’s home.
- Nurses must never work in isolation. The chemotherapy nurse must always work as part of a team, with robust lines of support, e.g. from medical and pharmacy colleagues.
  - Concurrent clinics provide readily accessible support.
  - In the absence of concurrent clinics, there must be clearly defined and accessible lines of support available (may be remote access, e.g. by telephone).
- Systems should be in place that permit nurses to admit a patient from the nurse-led clinic to a ward without involving a doctor—to avoid delays and meet the rapid-access requirements of acute oncology, e.g. for treatment of suspected neutropenic sepsis [6].
- A nurse may lead chemotherapy review in partnership with an appropriately skilled and experienced oncology pharmacist.
- When setting up a new nurse-led chemotherapy review service, involvement of staff who are keen and motivated will help to make it work.

## Conclusion

There is no single model for nurse-led chemotherapy review; however, UKONS proposes several core principles and features. In summary, these recommendations encompass the following issues:

- Nurse-led chemotherapy review is not a replacement for or a duplication of the medical clinic.
- The added value of nursing practice, e.g. holistic patient focus and support for families and carers, will underpin nurse-led chemotherapy review.
- Nurses should never work in isolation, and robust lines of support are essential for lead chemotherapy nurses.
- The nurses providing the service should have recognised qualifications and/or extensive competency-based experience in chemotherapy and clinical examination.
- Excellent communication skills are essential, as is commitment to information sharing across all aspects of the patient’s care pathway; completion of an advanced communication course is recommended.
- Electronic prescribing is a future minimum standard. Where it is not yet available, computer-generated prescribing is essential. The system should include a full toxicity record with agreed parameters.
- As with medical review, nurse-led chemotherapy review will require adequate administrative and in-clinic support.
- Nurse-led chemotherapy review will need to demonstrate its effectiveness in terms of both patient care/satisfaction and value for money.
- Nurse prescribing will become a gold standard in nurse-led chemotherapy review, and non-prescribers should consider completing an appropriate course.

## Clinic example 1. Nurse-led oral chemotherapy for lung cancer: Lanarkshire [34]

A nurse-led review clinic for patients with non-small-cell lung cancer who are scheduled to receive oral vinorelbine on day 8 of their chemotherapy cycle has been underway in Lanarkshire since 2007. The clinic was planned with the aim of improving the service for eligible patients through provision of oral versus intravenous vinorelbine and to improve capacity in the chemotherapy day unit and in pharmacy. The clinic is protocol driven, with clearly set-out inclusion and exclusion criteria (e.g. performance status and comorbidities), and clinic checklists (e.g. an algorithm for transfer to medical review). There is annual staff training and a stipulation of no deviation from the agreed regimens.

Two days before the oral vinorelbine dose is due, the consultant issues the prescription and blood samples are taken the following day. On the treatment day itself, the patient is telephoned by the nurse for an initial assessment, and the blood results are checked. If the treatment is to go ahead as planned, the patient is instructed to take the prescribed oral antiemetic 1 h before attending the clinic. On arrival, the nurse assesses the patient and ensures that all protocol requirements are met, before administering the oral vinorelbine. On the following day, the nurse telephones the patient to enquire about any after effects of the treatment.

After a pilot audit of 18 patients, it was concluded that the nurse-led methodology was practical and feasible, and replicable at other sites. The time patients spent in the clinic was reduced from an average of 4 h for intravenous vinorelbine to 30 min. In a postal patient satisfaction survey completed by 14 recipients, 100% said that the nurse was as thorough as the doctor, although 2/14 would have preferred to receive medical assessment. In general, the respondents felt that the process was safe and provided improved continuity of care compared with the previous service.

## Clinic example 2. Nurse-led chemotherapy nadir review clinic: West Cumbria [Helen Roe, personal report]

The chemotherapy nadir clinic for West Cumbria was established in 2002 with the primary aim of harnessing the knowledge and skills of a consultant cancer nurse (CCN) with prior experience of running such a service. At the same time, it was hoped that the clinic would help to reduce pressure on the oncologists’ hours. It is led and provided by the CCN, an independent practitioner and non-medical prescriber. The clinic forms one part of a team approach to the delivery of chemotherapy and the support of patients (including clinical trials participants) receiving oral or intravenous chemotherapy for breast cancer and a range of other tumours. The CCN is supported by a clinical oncologist based at a separate hospital, and the aseptic pharmacy team is on hand to provide advice when needed. All activity relating to the clinic is in the CCN’s name.

The CCN undertakes a holistic assessment of chemotherapy recipients between cycles, looking at toxicities experienced and at patients’ ongoing care needs in general. She can prescribe chemotherapy and supportive medications, delay or reduce the chemotherapy dose as required, and refer patients directly to other professionals. Through continual assessment of each patient, the CCN is also able to assess the response to treatment and recommend regimen changes in line with network-approved treatments, including access to new agents through the Cancer Drugs Fund. Between their scheduled visits, patients are able to contact the CCN and self-refer to her clinic.

On the day of treatment, patients are assessed by the nurses who will be delivering the chemotherapy, who will contact the CCN if they have any concerns. Again, the CCN is able to prescribe supportive medications and/or make changes to the chemotherapy as required, thereby avoiding the delays inherent in a system that requires a doctor’s prescription. As a principal investigator for some of the clinical trials underway locally, the CCN is also able to enter patients into studies with the support of the clinical trials team.

A study of the CCN-led clinic, in comparison with a similar clinic delivered by an oncologist, has concluded that patients reviewed by the CCN do not receive a lesser service [35]. Moreover, the nurse tends to identify more patient-focused concerns than her medical colleague.

The West Cumbria model enables continual assessment of patients and prompts effective management of post-chemotherapy symptoms. Pharmacy and nursing staff are aware of required changes prior to the day of treatment delivery, which helps to prevent wastage of chemotherapy agents and chair time. The model demonstrates how a team of professionals can work together to improve the patient experience of receiving chemotherapy.

## Clinic example 3. Community-based treatment: Lymington, Hampshire [Elaine Lennan, personal report]

Nurse-led chemotherapy review clinics have been established in Southampton for many years. They are run concurrently with the medical-led clinics and cover all tumour sites and treatment intentions. A recent initiative is the community-based service in Lymington, 20 miles from the cancer centre, which enables patient assessment and delivery of treatment closer to patients’ home, without attendance at the cancer centre.

Eligible patients are assessed by the nurse, either by phone or on the day of their clinic appointment. If the individual is deemed fit for chemotherapy, the nurse will prescribe the treatment (including dose reduction if required) and the supportive medicines. A different nurse will then administer the treatment.

Because the community-based service is small, compared with clinics at the cancer centre, the nurse is able to spend more time with the patient and offers additional support and advice.

## Figures and Tables

**Table 1:** Educational attainment for nurse-led chemotherapy review

Educational attainment	Essential/recommended
Recognised qualification and/or extensive competency-based experience in chemotherapy [19]	Essential
History taking	Essential
Physical examination	Recommended^a^
Advanced assessment skills (e.g. synthesizing history and physical assessment)	Essential
Completion of a recognised course in advanced communication skills	Recommended^a^
Non-medical prescribing	Recommended^a^

a UKONS regards these educational attainments as a gold standard for the future of nurse-led chemotherapy review
